# Evaluating Sacrococcygeal Teratoma in an Adult Female: A Case Report

**DOI:** 10.1155/cris/4512252

**Published:** 2025-06-02

**Authors:** Dikshanta Acharya, Aashis Poudel, Aashish Giri, Dinuj Shrestha, Rupesh Raut

**Affiliations:** Department of Surgery, Patan Academy of Health Sciences, Lalitpur, Nepal

**Keywords:** case report, sacrococcygeal region, teratoma

## Abstract

Sacrococcygeal teratomas are rare in adults, despite being common in infants. Adult presentations are often asymptomatic and may go undetected until complications arise. We report a case of a 20-year-old female with a long-standing sacral swelling. Imaging revealed a large, well-circumscribed mixed solid-cystic mass in the presacral region causing displacement of pelvic structures. Surgical excision of the tumor was done along with the surrounding rectal wall. Early surgical intervention is essential to prevent malignant transformation and optimize outcomes.

**Summary**

Sacrococcygeal teratomas are uncommon in adults, despite being common in infants. They possess serious diagnostic difficulties because of their asymptomatic nature and potential for malignancy. Malignant transformation can be prevented through early surgical excision emphasizing the importance of timely management when diagnosed.

## 1. Introduction

Sacrococcygeal teratomas (SCTs) are tumors containing tissues from more than one germ layer [1]. They are one of the most common congenital tumors in newborns and infancy, prevalent in approximately 1 per 20,000 to 40,000 births and more common in females [2]. SCTs are rare in adult patients. The adults are generally asymptomatic and diagnosis is often an incidental finding during a digital rectal examination or radiological investigations. This makes it hard for clinicians to diagnose sacrococcygeal teratoma (SCT). However, if the mass is large enough, symptoms, such as pain in the sacrococcygeal area, constipation, bladder dysfunction, and neurological symptoms like lower extremity numbness may occur [3]. Diagnosis mainly relies on clinical examination and imaging. The suggested first-line therapy for SCTs is total surgical excision [4]. An early excision is recommended because of the increased rate of malignancy with age [5].

We report a case of SCT in a 20-year-old female who was managed successfully with surgical excision to emphasize the significance of treating SCT in childhood to avoid malignant changes in adulthood. Our work has been reported as per the SCARE 2023 guidelines criteria [6].

SCTs are neoplasms composed of tissues derived from more than one germ layer [1]. They represent a common congenital neoplasm in neonates and infants, occurring in approximately 1 out of every 20,000–40,000 live births, with a higher incidence in females [2]. SCTs are rare in adult patients. The adults are generally asymptomatic, and diagnosis is frequently made incidentally during per rectal examination or through radiographic evaluation. This makes it hard for clinicians to diagnose SCT. However, if the mass is large enough, symptoms may include discomfort in the sacrococcygeal region, difficulty with defecation, urinary dysfunction, and neurologic manifestations such as numbness in the lower limbs [3]. Diagnosis is primarily based on a combination of physical examination and radiological imaging. The primary treatment of choice for SCTs is total surgical excision [4]. Early excision is recommended because of the increased rate of malignancy with age [5].

We present a case of SCT in a 20-year-old female who was managed successfully with surgical excision to emphasize the significance of treating SCT in childhood to avoid malignant changes in adulthood. We report this case as per the SCARE 2023 guidelines criteria [6].

## 2. Case Presentation

### 2.1. History

**Figure 1:**
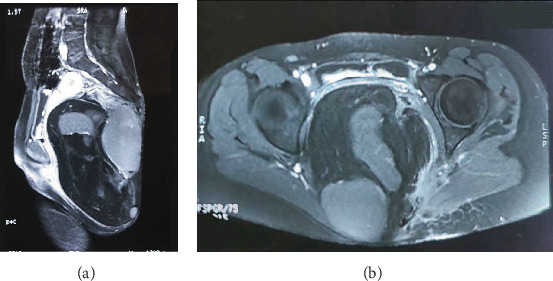
(a) Magnetic resonance imaging (MRI) showing a large well-defined mass with mixed solid and cystic component in lower sacral region. (b) MRI showing multiloculated cystic lesion causing anterior displacement of uterus.

A 20-year-old female presented to a hospital in Bihar, India with the complaint of swelling in her sacral region, which was around 10 × 5 cm in size, non-tender, and without any discharge. However, no interventions were done because of her pregnancy. She visited the same center 2 months after her delivery where she was thoroughly examined, and fine needle aspiration cytology from the swelling was done, which showed collagenous stroma, low cellularity, spindly nuclei arranged in short fascicles, and in whorls lacking cytological atypia without any evidence of inflammation, dysplasia, or malignancy. A contrast magnetic resonance imaging (MRI) of the pelvis was also done, which showed a large well-defined mixed solid and cystic hypointense soft tissue mass lesion in the midline pelvic cavity in the presacral region extending in the left gluteal region. (Figure 1) The lesion caused a significant mass effect leading to anterior displacement of the uterus and bladder. There was no obvious intraspinal extension or contrast enhancement in the lesion. The features were suggestive of a presacral mass, possibly a sacrococcygeal teratoma.

She visited Bharatpur Cancer Hospital after 3 days with the reports where an incisional biopsy was done. A small incision was made and a biopsy was taken. Suturing at the incision site was done and the patient was advised to alternate day dressing with follow-up after 14 days. The histopathology report showed squamous lining epithelium with acanthosis and elongated rete ridges in some areas, and the underlying dermis showed dense inflammatory infiltrates comprising lymphocytes, neutrophils, eosinophils, and plasma cells. There was mild edema with the proliferation of capillary-sized blood vessels without any atypia.

A month later, the patient had an increase in the size of swelling and discharge from the previous incision site. With these complaints, she presented to our neurosurgery OPD with these complaints. There was a small amount of discharge which was yellowish to light red in color and non-foul smelling. It was associated with intermittent dull aching pain. The bowel and bladder habits were normal and there was no history of fever, burning micturition, or past medical, surgical, drug, allergic, or personal history.

### 2.2. Examination

On local examination, the swelling over the sacral region measured about 12cm × 8 cm. The mass was soft, irregular, and non-fluctuant, and scar mark was present over the swelling. There was mild tenderness and whitish discharge from below the scar mark. There was no tingling or numbness, and power was 5/5 in both lower limbs.

### 2.3. Investigations and Imaging

Investigation of blood grouping and Rhesus factor typing and serology of Hepatitis B surface antigen, Hepatitis C Virus, and Human Immunodeficiency Virus 1 and 2, Prothrombin time/ International Normalized Ratio, platelet count, total leukocyte, differential count, random blood sugar, serum creatinine, C reactive protein, serum albumin, erythrocyte sedimentation rate (ESR), hemoglobin, and routine urine examinations were sent, which were within normal physiological limits.

**Figure 2:**
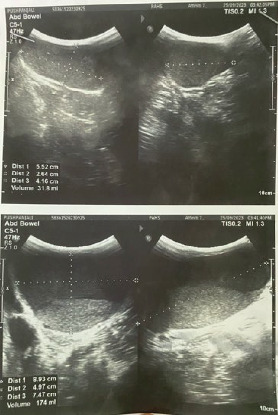
USG showing a well-defined, thin-walled, cystic lesion measuring 8.9 cm × 7.4 cm × 4.9 cm (~174 mL volume) with a low level of internal echoes noted in the subcutaneous plane in the midline in the lower sacral region.

 Ultrasonography (USG) of the lesion was done. (Figure 2) A provisional diagnosis of a benign soft tissue tumor of the sacrococcygeal region (likely a SCT) was made, and the patient was planned for excision of the SCT.

### 2.4. Treatment Plan

**Figure 3:**
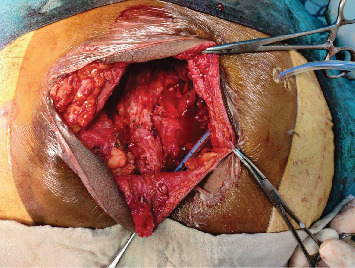
Visualization of mass intraoperatively.

**Figure 4:**
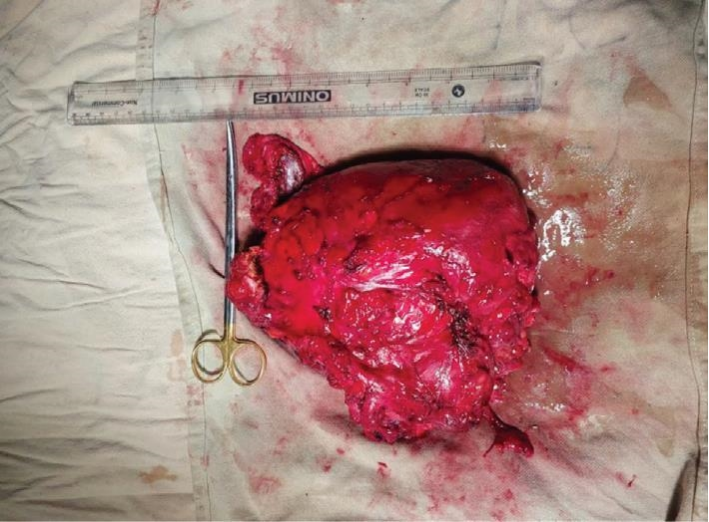
Sacrococcygeal mass after complete excision.

The patient was kept in the prone position and painting and draping was done, the attending neurosurgeon made a transverse incision over the swelling, and subcutaneous dissection was done. The capsule was identified, dissection was done around the capsule, and the median sacral artery was ligated. Dense adhesions of the tumor to the posterior rectal wall were seen, so the tumor was excised along with the adherent rectal wall. Primary repair of the rectum was done with polyglactin 2–0, the peritoneum was closed, the coccyx was removed, hemostasis was secured, and a drain was placed in the pelvic space and fixed suture. The wound was closed in layers and the excised specimen was sent for biopsy. After the surgery, the patient was shifted to the neurosurgery intensive care unit (Figures 3 and 4).

Postoperatively, hemoglobin was low so, four pints of whole blood were transfused. Noradrenaline was started due to low blood pressure, other than this vitals were within normal physiological limits. Noradrenaline was discontinued on the 4th postoperative day. After 1 week, the patient was transferred to the ward. On the 8th postoperative day, there was discharge from the wound, so the suture was removed for inspection. Wound dehiscence was seen, so a vacuum dressing was done for the next 2 weeks. The wound was healthy with granulation tissues, so the wound was left to heal via secondary intention.

### 2.5. Follow-up

After 2 week, the patient came for a follow-up with the pathological report of the tumor. The reports showed the presence of hairs, muscles, and fats as solid components and mucinous fluid with a cheese-like granular structure as a cystic component on cut section. On microscopic examination, the section from the mass showed derivatives from all the three germ cell layers. No immature component was identified. All the margins were unremarkable. The final diagnosis of mature teratoma was made which was negative for malignancy. She had a complaint of dysuria and following overflow urinary incontinence. The wound healing was present. On subsequent follow-up, the wound was healed completely however, the patient's urinary complaints were not completely improved.

## 3. Discussion

We reported a case of a 20-year-old female who presented with swelling on her sacral region which was present since birth. After a detailed history examination and investigations, it was found that the mass was a sacrococcygeal teratoma. Teratoma contains more than one germ layer with matured or immature elements. There are four different forms of SCT based on Altman's classification: (a) type I tumor with a hip deformity that primarily originates at the sacrococcygeal region; (b) type II tumors are predominantly external but extend into the pelvic cavity; (c)type III tumors are mostly intrapelvic with limited extension into the gluteal region; and (d) type IV tumors are entirely internal without exophytic extension [7].

Histologically they can be classified as mature teratomas, immature teratomas, and malignant teratomas. These teratomas can have solid, cystic, or mixed components. The pluripotent germ cells, located at Hensen's node in the coccygeal region, fail to migrate properly during development and proliferate to form SCT [5]. Our case was a mature teratoma containing derivatives of all three germ layers.

SCTs are typically identified prenatally as masses protruding from the caudal end of fetus or during infancy. It is often asymptomatic or presents with signs of obstruction to the rectum or urinary tracts. Rarely, affected infants may exhibit weakness, pain, or paralysis. In our case, the patient was asymptomatic at first even though there was anterior displacement of the uterus and bladder. SCT is the most prevalent germ cell neoplasm in children, occurring in approximately 1 per 20,000–40,000 live births [2]. SCTs rarely present in adulthood [5]. However, a few case reports have been recorded. Baikady and Singaram [8] reported a case of a 56-year-old female patient with swelling in the sacral region without any symptoms which was diagnosed as SCT on MRI. Similarly, Afuwape et al. [9] reported a case of a 24-year-old female with a history of recurrent pain in the gluteal region for 9 years, who was diagnosed with SCT.

Various imaging modalities can be used in the assessment of SCTs. X-rays of the pelvic and sacral region may show soft tissue opacities, congenital anomalies of the sacrum, calcific deposits, or sacral erosion, often accompanied by widening of the presacral space. Tumors arising directly from the sacrum can infiltrate and expand the sacrum. SCT produces anterior erosion of the sacrum and may present as a protruding extrinsic mass, containing teeth or bone. An intravenous urogram may show displacement of the bladder and one or both ureters, potentially indicating obstruction, while a barium enema may reveal an extrinsic pressure defect on the rectum. Bone scintigraphy may indicate elevated bone turnover, suggestive of either inflammatory or neoplastic conditions. USG shows lesion morphology, including dimensions and anatomical location while also assisting in the evaluation of hepatic metastasis or hydronephrosis. USG-guided fine-needle aspiration may facilitate cytological evaluation. Among imaging modalities, high-resolution computed tomography remains the most definitive tool for delineating anatomical structures with precision. In some cases, examination under anesthesia with biopsy may be necessary for histopathological confirmation; however, for more definitive diagnosis, excisional biopsy is often preferable. When malignancy is suspected, accurate diagnosis is essential to guide decisions regarding radical surgical resection or consideration for adjuvant radiotherapy. On gross inspection, teratomas are typically well-defined lobulated masses containing multiple structures, including mucus-filled cysts, nodules of cartilage and bone, and rarely teeth and hair. In the pineal region, these tumors are not usually invasive. For mature teratomas that can be completely excised, the 5-year survival rate is 80% – 90% [10].

On both Computed Tomography and MRI, teratomas are characterized by their striking heterogeneity in density and signal. Intratumoral cysts with well-defined margins and variable density or signal of contents (watery, proteinaceous, and lipid) are intermixed with calcifications or ossifications and areas of variegated soft tissue appearance, including hemorrhage [10].

Total surgical excision is the definitive and most effective treatment for SCT. The surgical approach is determined by tumor size, composition, and extent. The trans-sacral approach is optimal for tumors ≤10 cm situated below the third sacral vertebra, mainly Altman types I and II in the lower pelvic region [5, 7, 11].

SCT left untreated progresses into malignancy. The risk of malignant transformation in adult SCT was found to be around 6% [12]. Malignant transformation can occur in any of the components, with ectodermal squamous cell carcinoma being the most frequently observed type. Other malignancies include basal cell carcinoma, melanoma, adenocarcinoma, sarcoma, and thyroid carcinoma. The long-term treatment outcomes of the surgery are minimal, with few cases of constipation and occasional bed wetting which can be managed conservatively [13]. Dysuria and following overflow urinary incontinence is managed by urinary catheterization and medication with alpha-adrenergic antagonists or anti-cholinergics [13].

## 4. Conclusion

We reported an adult female with SCT which was an incidental finding on a routine checkup. Physical examination and radiological investigations help in the diagnosis of this condition. Considering its rarity, it is often missed in childhood and can undergo malignant transformation in adults. So, surgical excision must be done early to prevent malignancy.

## Data Availability

No datasets were collected or analyzed during the preparation of this case report. All relevant clinical and diagnostic data supporting the findings are included within the manuscript.
